# Catestatin prevents endothelial inflammation and promotes thrombus resolution in acute pulmonary embolism in mice

**DOI:** 10.1042/BSR20192236

**Published:** 2019-11-22

**Authors:** Hua Chen, Dongxia Liu, Lan Ge, Tao Wang, Zhenzhen Ma, Yuping Han, Yawei Duan, Xin Xu, Wei Liu, Jing Yuan, Jing Liu, Ruyi Li, Rongpin Du

**Affiliations:** 1 5th Department of Cardiology, Hebei General Hospital, Shijiazhuang 050051, P.R. China; 2 Science and Education Office, Hebei General Hospital, Shijiazhuang 050051, Hebei Province, P.R. China; 3 North China University of Science and Technology, Tangshan 063200, Hebei Province, P.R. China; 4 Department of Emergency, Hebei General Hospital, Shijiazhuang 050051, Hebei Province, P.R. China; 5 Department of Internal Medicine Nursing, Hebei College of Traditional Chinese Medicine, Shijiazhuang 050051, Hebei Province, P.R. China; 6 4th Department of Cardiology, Hebei General Hospital, Shijiazhuang 050051, Hebei Province, P.R. China; 7 Department of Endocrinology, Hebei General Hospital, Shijiazhuang 050051, Hebei Province, P.R. China; 8 6th Department of Cardiology, Hebei General Hospital, Shijiazhuang 050051, Hebei Province, P.R. China

**Keywords:** catestatin, endothelial cells, inflammation, p38, thrombus formation, TLR-4

## Abstract

Catestatin (CTS), a catecholamine-release inhibitory peptide, exerts pleiotropic cardiac protective effects. Pulmonary embolism caused by deep vein thrombosis involving vascular dysfunction. The present study aims to investigate the effects of CTS on thrombus formation that may inhibit the development of pulmonary embolism and its potential pathway. Acute pulmonary embolism (APE) model was developed as an *in vivo* model. The effects of CTS on mice with APE were examined. Human pulmonary artery endothelial cells (HPAECs) were pretreated with CTS before thrombin stimulation, and endothelial inflammation and underlying mechanisms were evaluated *in vitro*. That plasma CTS level was decreased in APE mice, while the number of platelets was significantly increased. The decreased circulating CTS level negatively associated with the number of platelets. CTS administration increased the survival rate of APE mice and protected against microvascular thrombosis in lung. APE-induced the increase in platelets number and plasma von Willebrand factor (VWF) were inhibited by CTS. Platelets from CTS-treated APE mice showed impaired agonist-induced platelets aggregation and spreading. CTS also ameliorated APE-induced the systemic inflammatory response. In *in vivo* study, thrombin-induced the increase in inflammation, TLR-4 expression and p38 phosphorylation were abrogated by CTS in HPAECs. Furthermore, TLR-4 overexpression inhibited the effect of CTS on VWF release and inflammation in HPAECs. Collectively, CTS increases thrombus resolution by attenuating endothelial inflammation at partially via inhibiting TLR-4-p38 pathway. The present study may provide a novel approach for anti-thrombosis.

## Introduction

Platelets aggregation and subsequent hemostatic plug formation result in primary hemostasis upon vascular injury [1,2]. In arterial diseases, occlusive platelets thrombus induced by platelets aggregation is one of the major causes of myocardial infarction, stroke and pulmonary arterial hypertension [3–6]. It has been recognized that endothelial inflammation is a critical risk factor for arterial thrombotic diseases [2,6]. Endothelial cells link blood and vessels, and play an important role in thrombo-inflammation during thrombosis [1,7]. Damage to vascular endothelium leads to exocytose Weibel–Palade storage bodies and blood flow stagnation by prolonged immobility [8]. The releases of multimeric glycoprotein von Willebrand factor (VWF) and adhesion molecules P-selectin and E-selectin from endothelial cells cause platelets and leukocytes recruitment, respectively [1]. Furthermore, VWF binds with collagen to the platelets surface for thrombin generation and inflammatory cells releases, such as neutrophils, macrophages and mast cells [9–11]. This series of thrombo-inflammatory events eventually lead to vein occlusion and death by thrombosis.

Catestatin (CTS), a 21-amino-acid-residue peptide, is generated endogenously from the neuroendocrine hormone chromogranin A [12,13]. A substantial line of evidences demonstrate that CTS is a pleiotropic modulator against vascular diseases, such as antihypertensive, vasodilator and angiogenic effects [14–16]. CTS suppresses smooth muscle cell proliferation, collagen production, macrophage inflammatory response and myocardial cell apoptosis, and ameliorates heart failure and myocardial infarction [17–19]. Recently, a previous study showed that CTS is a novel anti-atherogenic peptide via inhibiting inflammatory response in endothelial cells [20]. This study together with the potential role of CTS in inhibiting endothelin-1 release and promoting nitric oxide generation indicate that CTS may exert beneficial effects in the endothelium [21,22].

TLR4 is a membrane receptor that exerts its actions through p38 mitogen-activated protein kinase (MAPK) [23–25]. It has been shown that TLR-4 is an essential factor in infection-related thrombosis [26]. Moreover, during arterial thrombosis, TLR-4 level is up-regulated via p38 MAPK activation [27]. Here, our present study evaluated the effects of CTS on inflammatory responses and thrombus formation in a model of acute pulmonary embolism (APE). The results showed that CTS attenuates endothelial cell inflammation via TLR-4-p38 pathway which may contribute to its effective anti-thrombotic activity *in vivo*.

## Materials and methods

### Materials and reagents

CTS was purchased from KareBay Biochem (Monmouth Junction, New Jersey, U.S.A.). Antibodies against phospho-p38 (P-p38), p38, TLR-4 and GAPDH were obtained from Cell Signaling Technology (Danvers, MA, U.S.A.). EGM-2 medium, fetal bovine serum (FBS), penicillin and streptomycin were purchased from Invitrogen (Carlsbad, CA, U.S.A.). P38 MAPK inhibitor SB203580 was obtained from Sigma (St. Louis, MO, U.S.A.). TLR-4 blocker CLI-095 was from InvivoGen (Carlsbad, CA, U.S.A.).

### APE model and CTS administration

Eight-week-old C57/BL6 mice were purchased from the Animal Experimental Center of Hebei Medical University (Hebei, China). The mice experiments were performed in the Department of Cardiology of Hebei General Hospital. All animal experimental procedures complied with the guidelines by the Association for Assessment and Accreditation of Laboratory Animal Care International and approved by the Institutional Animal Ethics Committee of Hebei General Hospital (HGHDW2017-0042). Mice were randomly divided into the following groups: control (*n*=20), control CTS (*n*=20), APE (*n*=20), and APE CTS (*n*=20). The mice were anesthetized in a chamber containing 2% isoflurane mixed with 0.2 l/min 100% O_2_ and maintained with a face mask (0.5% isoflurane). CTS (2.9 μg/g body weight) was slowly injected into the inferior vena cava 30 min before challenge. The dose of CTS was chosen on the basis of a previous study [28]. Acute experimental pulmonary embolism was induced by injection of a mixture of 0.8 mg/kg collagen and 60 μg/kg epinephrine through the inferior vena cava. Animals that were still alive 30 min after the challenge were considered to be survivors.

### Peripheral blood analysis

Peripheral blood was collected via cardiac puncture 30 min after APE challenge using a 1-ml heparinized syringe. A total of 100 μl aliquots were immediately sampled and analyzed using a veterinary Hematrue hematology bench top analyzer (Heska Corporation Loveland, Colorado, U.S.A.). The number of platelets was measured. Commercial ELISA kits were used to determine CTS (CUSABIO, Cedarlane, Burlington, Canada), P-selectin, E-selectin, MCP-1, (R&D Systems, Minneapolis, MN, U.S.A.), MPO and VWF levels (Abcam, Shanghai, China).

### Histological analysis

Thirty minutes after APE challenge, the lungs were fixed *in situ* by intratracheal instillation of paraformaldehyde, embedded in paraffin, and sectioned at 5-μm thickness. The slides were deparaffinized in xylene and rehydrated by 100, 95, 70 and 50% ethyl alcohol in succession. Afterward, the sections were stained with Hematoxylin and Eosin and imaged with a light microscope (BX5, Olympus, Tokyo, Japan).

### Platelets isolation and functional assays

Platelet-rich plasma (PRP) at the concentration of 3.5 × 10^8^ platelets/ml was obtained from whole blood by mixing with 3.8% sodium citrate (volume ratio of 9:1). After centrifugation at 1000×***g*** for 10 min, the PRP was further centrifuged at 120×***g*** for 7 min to remove residual erythrocytes. Aggregation of PRP induced by thrombin (1 U/ml) was measured at 37°C while stirring using a 2-channel aggregometer (Chrono−log Model 590−2D, Chromo−Log Corp., Havertown, PA, U.S.A.). For platelets spreading assay, washed platelets were placed on fibrinogen (100 μg/ml)-coated coverslips and stimulated with thrombin (0.01 U/ml) or ADP (10 μmol/l) for 45 min at 37°C. Platelets were fixed with 4% formaldehyde for 10 min at room temperature and labeled with TRITC–conjugated Phalloidin. Images were captured with a laser confocal microscope (FV1000, Olympus, Tokyo, Japan) and cell area was quantified using ImageJ software (version 1.41, NIH, Maryland, U.S.A.).

### Cell culture

Human pulmonary artery endothelial cells (HPAECs) were obtained from Life Technologies Corporation (Thermo Fisher Scientific, Waltham, MA) and maintained in EGM-2 medium containing 10% FBS, 100 U/ml penicillin and 100 U/ml streptomycin in a 37°C incubator with 5% CO_2_.

### ELISA

The VWF (Abcam), P-selectin and E-selectin (R&D Systems) in endothelial cell supernatant were determined by ELISA, following the manufacturer’s instructions strictly.

### Adenovirus infection of HPAECs

Recombinant adenovirus encoding TLR-4 cDNA (AdTLR-4) was purchased from Sunbio Medical Biotechnology (Shanghai, China). Adenoviral infection of the HPAECs was performed at 50 multiplicity of infection (MOI) and treated with thrombin according to the experiment requirements. Lacz (Clonetech, CA, U.S.A.) acted as a negative control.

### Western blot analysis

HPAECs were harvested and lysed in RIPA lysis buffer containing a protease inhibitor cocktail (Thermo, MA, U.S.A.) and the protein was isolated according to the manufacturer’s instructions. The protein content was determined using a micro BCA kit (Beyotime). The samples containing equal protein (100 μg) were diluted in loading buffer and heated for 10 min at 99°C, followed by separation on 8% SDS/PAGE. The separated proteins were then transferred on to polyvinylidene fluoride membranes (Millipore, MA, U.S.A.). The membranes were blocked with 5% non-fat dry milk for 1 h at room temperature and incubated with antibodies against P-p38, p38, TLR-4 (1:500) and GAPDH at 4°C overnight. On the second day, the membranes were incubated with horseradish peroxidase–conjugated secondary antibody (1:1000, Beyotime, Shanghai, China). Blots were visualized using enhanced chemiluminescence (Thermo) and band intensities were quantified with ImageJ software.

### Statistical analysis

The results were obtained from at least four independent experiments and presented as mean value ± standard error of mean (SEM). *n* value represented the number of replicates and was mentioned in figure legends. No animal were excluded from analysis. The correlation between CTS level and platelets number was examined by the Pearson’s correlation test. Statistical analysis of data from two groups was performed using two-tailed Student’s *t* test. Data from more than two groups were compared by one-way ANOVA, followed by the Bonferroni’s multiple comparison test. A *P*-value less than 0.05 was considered statistically significant.

## Results

### Decreased blood plasma CTS level correlated with platelets number in a mouse APE model

**Figure 1:**
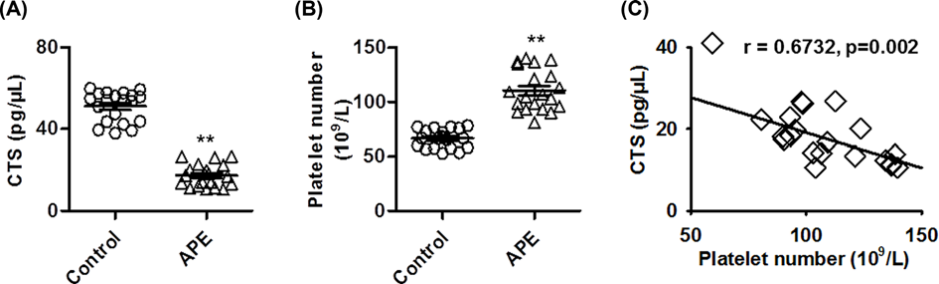
Circulating CTS level negatively correlates with platelets number in a mouse APE model (**A,B**) APE model was established by injection of a mixture of collagen and epinephrine through the inferior vena cava. Plasma CTS level (A) and platelets number (B) were measured, respectively. **P<0.01 vs. control, *n*=20/group. (**C**) Correlation analysis of the relationship between CTS level and platelets number by using Pearson correlation test (r = 0.6732).

As displayed in Figure 1A, compared with control group, the level of CTS was significantly decreased after APE challenge. Expectedly, the number of platelets was markedly higher in APE group than in control group (Figure 1B). Moreover, we analyzed the possible correlation between CTS level and the number of platelets. The results showed that the decreased CTS level was negatively correlated with platelets number with the correlation coefficients of 0.6732 (Figure 1C). These data suggest that CTS level is reduced during thrombosis and correlated with platelets number.

### CTS administration decreased the death rate and pulmonary hemorrhage induced by APE

**Figure 2:**
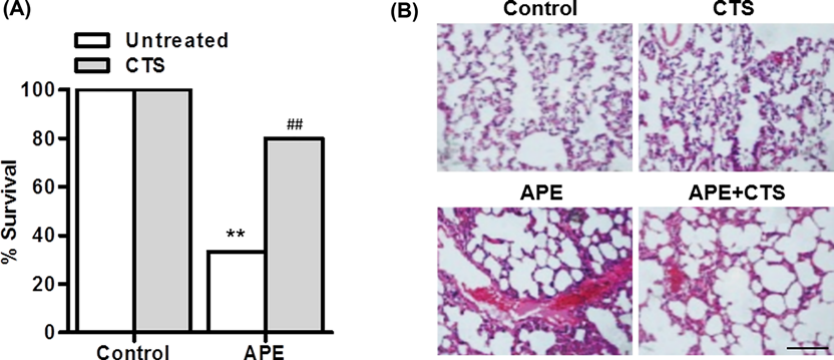
CTS protects against APE-induced microvascular thrombosis (**A**) Mice were injected with CTS for 30 min before APE challenge. The survival rate was calculated. **P<0.01 vs. control; ^##^*P*<0.01 vs. APE, *n*=20/group. (**B**) Representative sections of H&E-stained lungs from control, CTS-treated, APE, and CTS-treated APE mice. Scale bar, 100 μm.

To investigate the effect of CTS on thrombus, control or APE mice were received CTS treatment. Figure 2A showed that APE mice with the exception of three (3/10) died within 30 min of administration of collagen and epinephrine. In contrast, CTS–treated APE mice showed a significant increase in the number of survivors (8/10). We further examined whether CTS decreased the death rate via inhibiting thrombus formation. Mice from the APE group showed marked pulmonary embolization, damaged alveolar walls and inflammatory cell infiltration. However, changes such as pulmonary hemorrhage and inflammatory cell infiltration were attenuated in CTS-treated APE mice (Figure 2B).

### CTS displayed effective anti-thrombotic activity in APE mice

**Figure 3:**
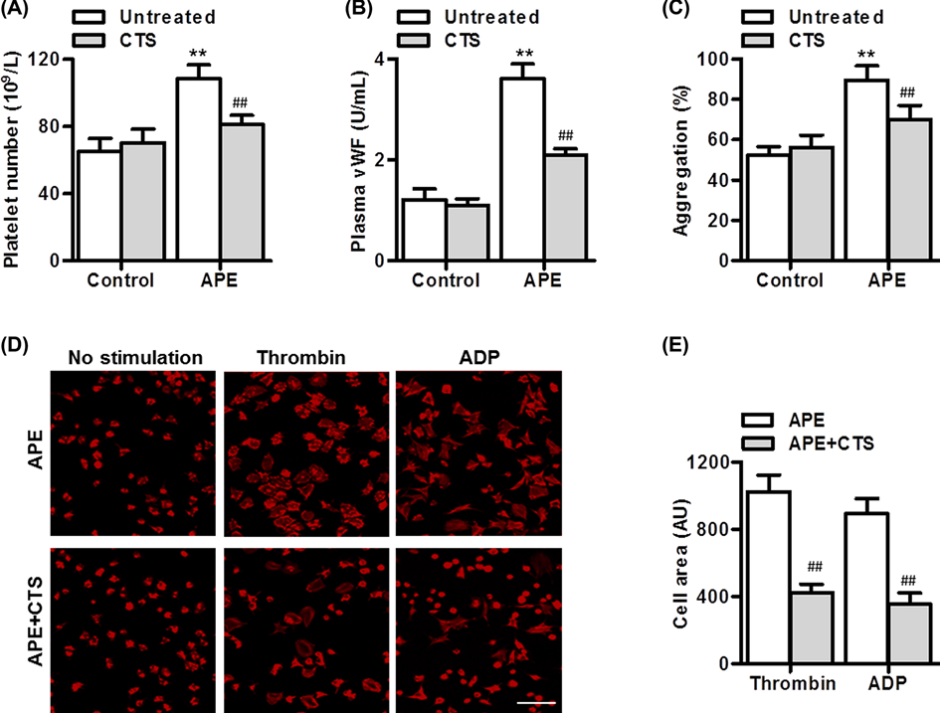
CTS impairs APE-induced thrombus formation (**A,B**) Thirty minutes after APE challenge, plasma platelets number (A) and VWF level (B) were measured. (**C**) Thrombin (1 U/ml)-induced platelets aggregation were measured using an aggregometer. (**D,E**) Platelets were allowed to spread on fibrinogen-coated coverslips in the presence of thrombin (0.01 U/ml) or ADP (10 μM) for 45 min, fixed, and stained with TRITC-phalloidin. Representative images were shown. Scale bar, 10 μm. The results were quantified using ImageJ software. **P<0.01 vs. control; ^##^*P*<0.01 vs. APE, *n*=6–10/group.

We next investigated the *in vivo* anti-thrombotic activity of CTS. Consistent with the decreased pulmonary hemorrhage, the number of platelets was significantly inhibited by CTS treatment (Figure 3A). Moreover, plasma VWF level analyzed by ELISA was remarkably increased in APE-treated mice, but this increase was blocked after CTS administration (Figure 3B). Similarly, APE challenge significantly potentiated thrombin-induced platelets aggregation, which was attenuated in CTS-treated APE mice (Figure 3C). Furthermore, in the absence of agonists, platelets showed similar spreading on fibrinogen in APE mice treated with or without CTS. However, in response to thrombin and ADP, CTS significantly reduced the spreading of platelets (Figure 3D,E).

### CTS impaired systemic thrombo-inflammatory responses induced by APE

**Figure 4:**
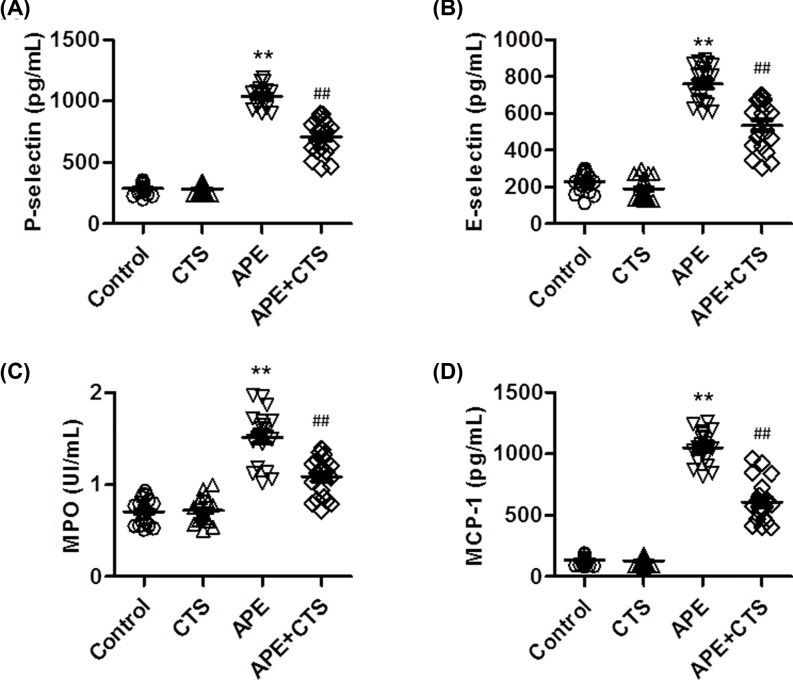
CTS attenuates APE-induced thrombo-inflammatory responses (**A**) After APE challenge, the plasma level of P-selectin (A), E-selectin (**B**), MPO (**C**) and MCP-1 (**D**) were determined by ELISA kits. **P<0.01 vs. control; ^##^*P*<0.01 vs. APE, *n*=10–12/group.

It is well known that inflammation is an established risk factor for arterial thrombotic diseases, along with greatly increased level of inflammatory cytokines [7]. As shown in Figure 4A,B, APE challenge significantly increased the level of P-selectin and E-selectin in blood, indicating that the increased release of adhesion molecules from endothelial cells may result in the deposition of platelets. However, CTS treatment markedly inhibited the increase in the level of P-selectin and E-selectin induced by APE. Moreover, APE also increased the level of MPO and MCP-1 in blood, which was inhibited in CTS-treated mice (Figure 4C,D). This suggests that CTS also inhibited APE-induced the release of inflammatory neutrophils and macrophages.

### CTS attenuated thrombin-induced endothelial inflammation by inhibiting TLR-4-p38 signaling

**Figure 5:**
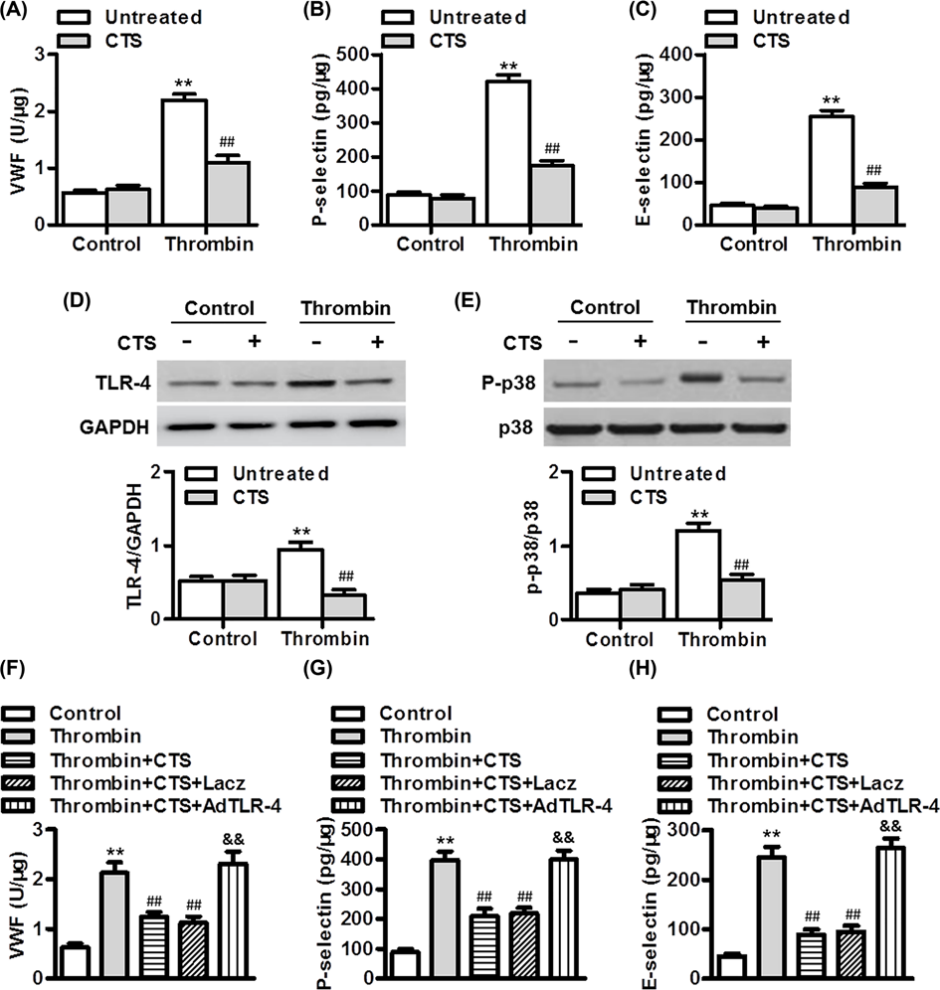
CTS ameliorates thrombin-induced endothelial inflammation via TLR-4-p38 signaling (**A**) HPAECs were pretreated with CTS (50 nmol/l) for 30 min and then stimulated with thrombin (1 U/ml) for another 12 h. The levels of VWF (A), P-selectin (**B**), and E-selectin (**C**) in endothelial cell supernatant were determined by ELISA. (**D,E**) The protein expression of TLR-4 (D) and the phosphorylation of p38 (E) were determined by Western blotting. **P<0.01 vs. control untreated; ^##^*P*<0.01 vs. thrombin untreated, *n*=6. (**F**–**H**) The CTS-pretreated cells were treated with thrombin and Lacz adenovirus or TLR-4 adenovirus (AdTLR-4, 50 MOI) for 24 h. The levels of VWF (F), P-selectin (G), and E-selectin (H) in endothelial cell supernatant. **P<0.01 vs. control; ^##^*P*<0.01 vs. thrombin; ^&&^*P*<0.01 vs. thrombin+CTS, *n*=6.

Given that endothelial cells play an important role in thrombo-inflammation and hemostasis, the effects of CTS on endothelial inflammation induced by thrombin was investigated *in vitro*. Similar to the *in vivo* results, CTS significantly inhibited the level of VWF in HPAECs induced by thrombin (Figure 5A). In addition, thrombin-induced the increase in endothelial P-selectin and E-selectin level was abrogated by CTS treatment (Figure 5B,C). The results of Western blotting showed that thrombin markedly increased the expression of TLR-4 and the phosphorylation of p38, which were all inhibited by CTS treatment (Figure 5D,E). To explore the association between TLR-4 and p38 signaling in mediating thrombin-dependent endothelial inflammation, we employed pharmacological inhibitors of TLR-4 and p38, and then measured their effects on each other signaling pathway activation, respectively. Thrombin-induced increase in p38 phosphorylation was inhibited by TLR-4 inhibitor CLI-095, whereas inhibition of p38 had no effect on the increased expression of TLR-4 by thrombin (Supplementary Figure S1). These results indicate that TLR-4 may be upstream of p38 in the thrombin-mediated inflammatory response. Following overexpression of TLR-4, the inhibitory effect of CTS on VWF release in HPAECs was abrogated (Figure 5F). Moreover, the effect of CTS on thrombin-induced inflammatory response was dramatically reversed after TLR-4 overexpression (Figure 5G,H).

## Discussion

**Figure 6:**
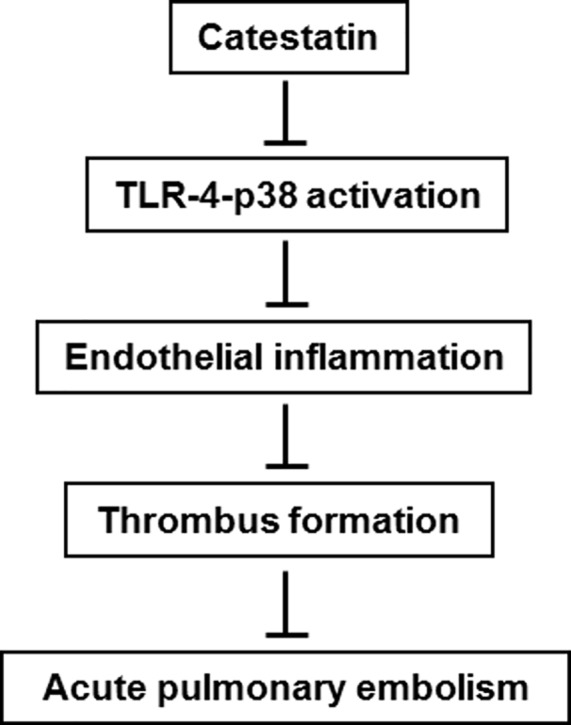
A schematic diagram explaining the anti-thrombotic effects of CTS

In the present study, we found that plasma CTS level is reduced in APE mice and promotes pulmonary hemorrhage as well as the development of thrombus formation. CTS treatment suppresses APE-induced pulmonary hemorrhage, platelets aggregation, and systemic inflammatory response. The beneficial effects of CTS involve inhibition of endothelial inflammation via TLR-4-p38 signaling pathway (Figure 6).

Thrombosis is the most common cause of death in the developed world [2]. Regarding to pulmonary arterial hypertension, pulmonary embolism is the major cause of pulmonary hypertension that is independent of heart disease or chronic lung disease [29–31]. It is well-known that embolizes into the pulmonary vasculature leads to APE, which mechanically obstructs pulmonary arteries and pulmonary hypertension [6,32]. CTS has been suggested to be implicated in multiple pathophysiological processes of cardiovascular diseases [14,17,20]. In vascular endothelium, CTS functions as a potent vasodilator and a pro-nitric oxide agent [21,22]. In addition, CTS is also involved in endothelial inflammation, response to endothelial dysfunction, and development of thrombosis-associated atherosclerosis [20,28]. Circulating CTS level was negatively correlated with the severity of atherosclerosis, which is characterized by chronic inflammation [20,28]. Similar to these previous studies, we showed that plasma CTS is also negatively associated with the increased number of platelets in APE mouse model, suggesting that the change of CTS level may be involved in thrombus formation. Accordingly, CTS significantly decreased the death rate of mice with APE and lung hemorrhage. Many studies employing thrombosis models have clearly have demonstrated the key role of VWF in thrombus formation [7]. VWF traps platelets and facilitating platelets aggregation [1]. We found that CTS reduced the circulating level of VWF induced by APE, and inhibited platelets aggregation and spreading, leading to thrombus resolution. It has been documented that CTS could inhibit TNF-α-induced inflammatory cytokines [20]. Consistently, our data showed that APE-induced the release of adhesion molecules, such as P-selectin and E-selectin, and infiltration of neutrophils and macrophages, such as MPO and MCP-1, were significantly attenuated by CTS. Given that inflammatory response plays a vital role in thrombus formation [1,7,8], we proposed that CTS may exert anti-thrombotic effects via its anti-inflammation properties.

It has been reported that TLR-4 pathway activation is associated with vasoconstriction and thrombus formation following endothelial injury [26]. TLR-4, a transmembrane signaling receptor, exerts its function via p38-dependent MAPK [23–25]. Multiple studies have shown the involvement of TLR-4 in thrombosis-associated ischemic diseases, including stoke, myocardial remodeling and atherosclerosis [33,34]. In the current study, we found activation of TLR-4 and p38 signaling after thrombin stimulation, suggesting the involvement of TLR-4 and p38 in thrombin-mediated endothelial inflammation. CTS treatment inhibited the activation of TLR-4 and p38, indicating that CTS ameliorates endothelial inflammation by inhibiting TLR-4 and p38 signaling. Since TLR-4 and p38 seem to be involved in the same signaling pathway, the sequence of TLR-4 and p38 was tested. Our results showed that inhibition of TLR-4 with specific inhibitor could substantially block p38 phosphorylation, while the p38 inhibitor failed to influence on the increased expression of TLR-4. These results further confirm that TLR-4 is the upstream of p38 in the thrombin-induced response. Overexpression of TLR-4 abrogated CTS-mediated inhibition of endothelial inflammation. These data suggest that CTS attenuates thrombin-mediated endothelial inflammation at least partially via inhibiting TLR-4-p38 signaling activation.

In conclusion, the present study demonstrates for the first time that CTS plays an important role in thrombosis. The decrease of CTS subsequently causes inflammation, leading to thrombus formation. Restoration of CTS inhibits this process. Our findings may provide a novel biomarker for thrombosis and a new therapeutic approach for thrombosis treatment.

## Supplementary Material

Supplementary Figure S1

## Abbreviations

APE: acute pulmonary embolism
CTS: Catestatin
FBS: fetal bovine serum
HPAEC: human pulmonary artery endothelial cell
MAPK: mitogen-activated protein kinase
PRP: platelet-rich plasma
P-p38: phospho-p38
VWF: von Willerbrand factor
