# Supplementary material for: Catestatin prevents endothelial inflammation and promotes thrombus resolution in acute pulmonary embolism in mice
Source: Biosci Rep. 2019 Nov 22;39(11):BSR20192236. doi: 10.1042/BSR20192236 (PMC6879352; doi:10.1042/BSR20192236)
Supplement: Supplementary Figure S1 [file BSR-2019-2236_supp.pdf]

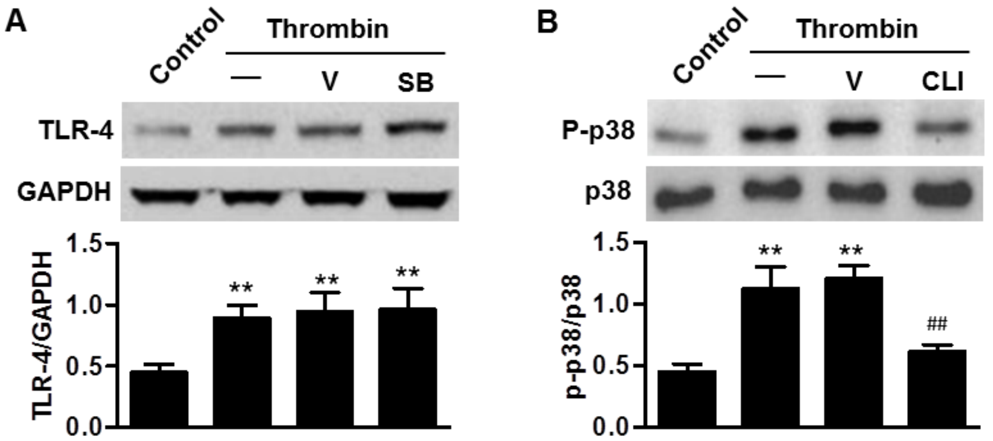

Figure S1. TLR-4 is the upstream of p38 in the thrombin-mediated inflammatory response. (A and B) The HPAECs were pretreated with p38 inhibitor SB203580 (SB, 10 μmol/L) (A) or TLR-4 blocker CLI-095 (CLI, 1 μmol/L) (B) for 30 min and then stimulated with thrombin (1 U/mL) for another 12 h. The protein expression of TLR-4 (A) and the phosphorylation of p38 (B) were determined by western blotting. **P<0.01 vs. control; ##P<0.01 vs. thrombin, n=6.
